# Growth dysregulation and p53 accumulation in human primary colorectal cancer

**DOI:** 10.1038/sj.bjc.6690464

**Published:** 1999-06

**Authors:** D S Watson, I Brotherick, B K Shenton, R G Wilson, F C Campbell

**Affiliations:** Department of Surgery, The Medical School, University of Newcastle, Framlington Place, Newcastle upon Tyne NE2 4HH, UK; Department of Surgery, South Cleveland Hospital, Cleveland, UK

**Keywords:** colorectal cancer, p53, apoptosis, proliferation, flow cytometry, immunohistochemistry

## Abstract

p53 accumulation is common in colorectal cancer, but effects on growth homeostasis are unclear. In this study, DNA content, cell cycle phase fractions and DNA strand-breaks consistent with apoptosis were assessed by flow cytometry in 42 fresh primary colorectal tumours and matched normal mucosa. p53 accumulation was assessed in 37 fixed tumour sections, by immunohistochemistry. In normal mucosa, 10.3 ± 6.6% (mean ± s.d.) cells were in DNA synthesis phase while 28.7 ± 17.9% showed apoptosis. A relationship suggestive of growth homeostasis, was observed between these parameters (*r* = 0.8; *P* < 0.05). In cancers, a greater number of cells were in DNA synthesis phase (15.6 ± 12.9% tumour vs mucosa 10.3 ± 6.6%; *P* < 0.02) while fewer showed apoptosis than normal mucosa (18.5 ± 17.0% tumour vs mucosa 28.7 ± 17.9%; *P* < 0.01). DNA synthesis and apoptosis fractions were unrelated in cancers, suggesting growth dysequilibrium. p53 accumulation was detected in 59% (22/37) tumours and was associated with reduced apoptosis compared to p53-negative tumours or mucosa (14.8 ± 15% p53 accumulation vs 26.3 ± 18% p53-negative; *P* < 0.05; vs 28.7 ± 17.9% mucosa; *P* < 0.05). p53 accumulation was unrelated to DNA synthesis phase fractions. p53 accumulation is accompanied by reduced apoptosis which may accentuate growth dysequilibrium in colorectal cancer. © 1999 Cancer Research Campaign


*British Journal of Cancer* (1999) **80**(7), 1062–1068
© 1999 Cancer Research Campaign
Article no. bjoc.1998.0464

### Growth dysregulation and p53 accumulation in human primary colorectal cancer

**DS Watson[1], I Brotherick[1], BK Shenton[1], RG Wilson[1,2] and FC Campbell[1]**

[1]Department of Surgery, The Medical School, University of Newcastle, Framlington Place, Newcastle upon Tyne NE2 4HH, UK; [2]Department of Surgery, South Cleveland Hospital, Cleveland, UK

**Summary** p53 accumulation is common in colorectal cancer, but effects on growth homeostasis are unclear. In this study, DNA content, cell cycle phase fractions and DNA strand-breaks consistent with apoptosis were assessed by flow cytometry in 42 fresh primary colorectal tumours and matched normal mucosa. p53 accumulation was assessed in 37 fixed tumour sections, by immunohistochemistry. In normal mucosa, 10.3 ± 6.6% (mean ± s.d.) cells were in DNA synthesis phase while 28.7 ± 17.9% showed apoptosis. A relationship suggestive of growth homeostasis, was observed between these parameters ($r = 0.8$; $P < 0.05$). In cancers, a greater number of cells were in DNA synthesis phase (15.6 ± 12.9% tumour vs mucosa 10.3 ± 6.6%; $P < 0.02$) while fewer showed apoptosis than normal mucosa (18.5 ± 17.0% tumour vs mucosa 28.7 ± 17.9%; $P < 0.01$). DNA synthesis and apoptosis fractions were unrelated in cancers, suggesting growth dysequilibrium. p53 accumulation was detected in 59% (22/37) tumours and was associated with reduced apoptosis compared to p53-negative tumours or mucosa (14.8 ± 15% p53 accumulation vs 26.3 ± 18% p53-negative; $P < 0.05$; vs 28.7 ± 17.9% mucosa; $P < 0.05$). p53 accumulation was unrelated to DNA synthesis phase fractions. p53 accumulation is accompanied by reduced apoptosis which may accentuate growth dysequilibrium in colorectal cancer.

**Keywords:** colorectal cancer; p53; apoptosis; proliferation; flow cytometry; immunohistochemistry

An equilibrium between cell gain via mitosis and loss by apoptosis influences structure, pattern and function of many tissues, but may become deranged in cancer. While mutations, rearrangements and deletions of specific growth regulatory genes are prerequisite to malignant change (Fearon and Vogelstein, 1990), the principal abnormalities leading to loss of growth homeostasis in colorectal cancer are unclear. The p53 protein is active as a transcription factor (Bargonetti et al, 1991) and exerts G1 checkpoint control (Kastan et al, 1991; El-Deiry et al, 1993) or initiates apoptosis (Shaw et al, 1992), particularly in response to DNA damage (Clarke et al, 1993). Functional inactivation of p53 promotes genomic instability (Bischoff et al, 1990) and is commonly, though not universally, associated with intracellular p53 protein accumulation (Hall and Lane, 1994; Save et al, 1998). p53 accumulation is detectable in many colorectal cancers (Purdie et al, 1991; Scott et al, 1991), with 40–60% having immunologically detectable levels (Kang et al, 1997; Ahnen et al, 1998). However, effects on growth homeostasis are unclear.

Previous studies investigated relationships between p53 overexpression, proliferation and apoptosis in colorectal cancer by in situ techniques viz immunohistochemistry, in-situ TUNEL assay of apoptosis, or histological assessment of apoptotic bodies, in fixed tissue sections. p53 overexpression was found to be associated with a lower apoptotic index (Kobayashi et al, 1995). In support of this finding, p53-negative tumours from patients aged less than 45 years were found to have higher apoptotic body counts (Langlois et al, 1997). However, Tsujitani et al (1996) reported converse findings in 67 colorectal tumours and found no correlation between p53 expression and apoptosis. Two studies found no relationship between p53 overexpression and Ki-67 expression (Lanza et al, 1996; Tsujitani et al, 1996).

*Received 20 August 1998*
*Revised 30 November 1998*
*Accepted 9 December 1998*

*Correspondence to*: FC Campbell

While histological assay of cell cycle antigens, apoptotic bodies or other in-situ assays of apoptosis in paraffin-embedded tissues are useful, they provide subjective and semiquantitative data. Interpretation differences could account for some variance in the previous reports. Optimal analysis of proliferative indices may be obtained from the examination of whole cell preparations from unfixed tissues, by flow cytometry (Shankey et al, 1993). This technique provides objective measurement of cell cycle phase fractions and may also use a modification of the TUNEL assay to assess apoptosis. In the present study, these methods were used to assay cell cycle phase distribution and apoptosis, in 42 fresh primary colorectal tumours and matched normal mucosa samples. p53 accumulation was assayed in fixed sections by immunohistochemistry. We sought to test the hypothesis that p53 accumulation in colorectal cancer is accompanied by disturbance of growth homeostasis. To test this hypothesis, we investigated relationships between p53 accumulation, proliferation, apoptosis, in colorectal cancers and in uninvolved mucosa.

### PATIENTS AND METHODS

### Retrieval of tissue

Fresh mucosal and tumour specimens were obtained immediately after colectomy from 42 consecutive patients with colorectal carcinoma, who were recruited from South Cleveland Hospital, Middlesbrough, the Royal Victoria Infirmary, Newcastle upon Tyne and some from Ninewells Hospital, Dundee. No patients had received preoperative radio- or chemotherapy. All patients gave

**Table 1** No relationship found between p53 accumulation, DNA ploidy, DNA S phase fraction or Dukes' stage of colorectal cancer (*P* = NS)

| | p53 accumulation | | Diploid (*n*) | Aneuploid (*n*) | DNA S phase (mean% ± s.d.) | Dukes' stage: (*n*) | | |
|---|---|---|---|---|---|---|---|---|
| | *n* | % | | | | A | B | C |
| Positive | 22 | 59 | 16 | 6 | 15.7 ± 14 | 1 | 14 | 7 |
| Negative | 15 | 41 | 10 | 5 | 14.8 ± 13 | 2 | 8 | 5 |

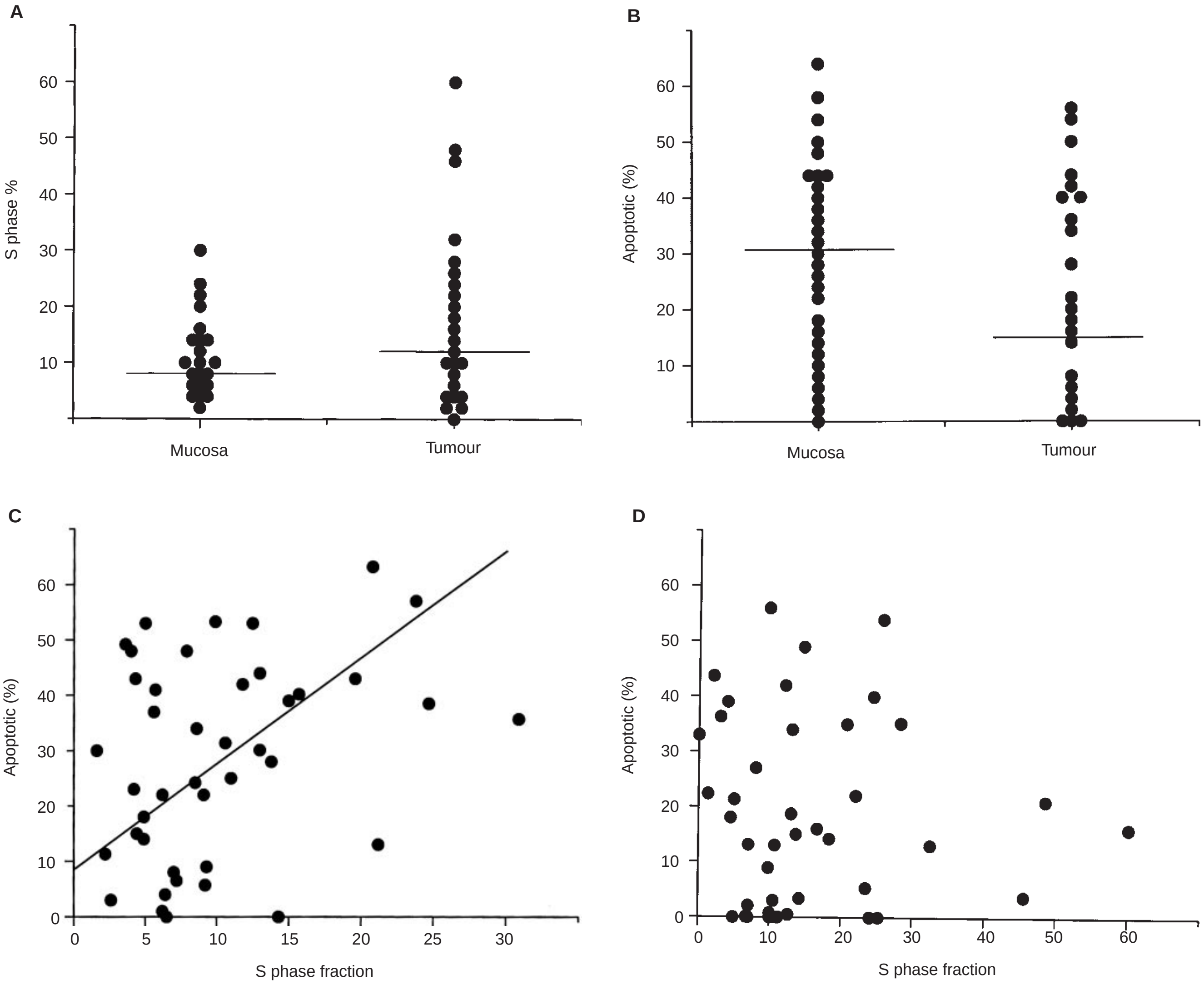

**Figure 1** (**A**) DNA synthesis (S) phase fractions of colorectal mucosal and tumour specimens. *P* < 0.02 by Wilcoxon matched pairs signed-rank test. Horizontal bar indicates median. (**B**) Percentage of cells showing apoptosis by TUNEL assay, in normal mucosa and colorectal tumours. *P* < 0.01 by Wilcoxon matched pairs signed-rank test. Median shown by horizontal bar. (**C**) Relationship between apoptosis and DNA S phase fraction in normal mucosa. (*r* = 0.8; *P* < 0.05 by Pearson test). (**D**) Percentage of cells showing apoptosis and proliferation in colorectal cancers. No relationship was observed informed consent to the study. Clinical data, including symptomatic presentation, age, family history, site of tumour and operation type were recorded. Tumour samples of approximately 1 g were removed without compromise to histopathological assessment. Normal mucosa was isolated from the same surgical specimens, distant from the tumours. Samples were then labelled and snap-frozen in liquid nitrogen, to enable transport to the laboratory. Colectomy specimens underwent histopathological assessment of differentiation, depth of invasion, lymph node metastases and resection margins. Blocks of fixed tumour were mounted in paraffin and sections cut for immunohistochemistry.

### Preparation of primary colorectal tumour tissue

Specimens of matched mucosa and tumour were rapidly thawed, finely minced, placed in Isoton II (Coulter Electronics, Luton,

*© 1999 Cancer Research Campaign* 

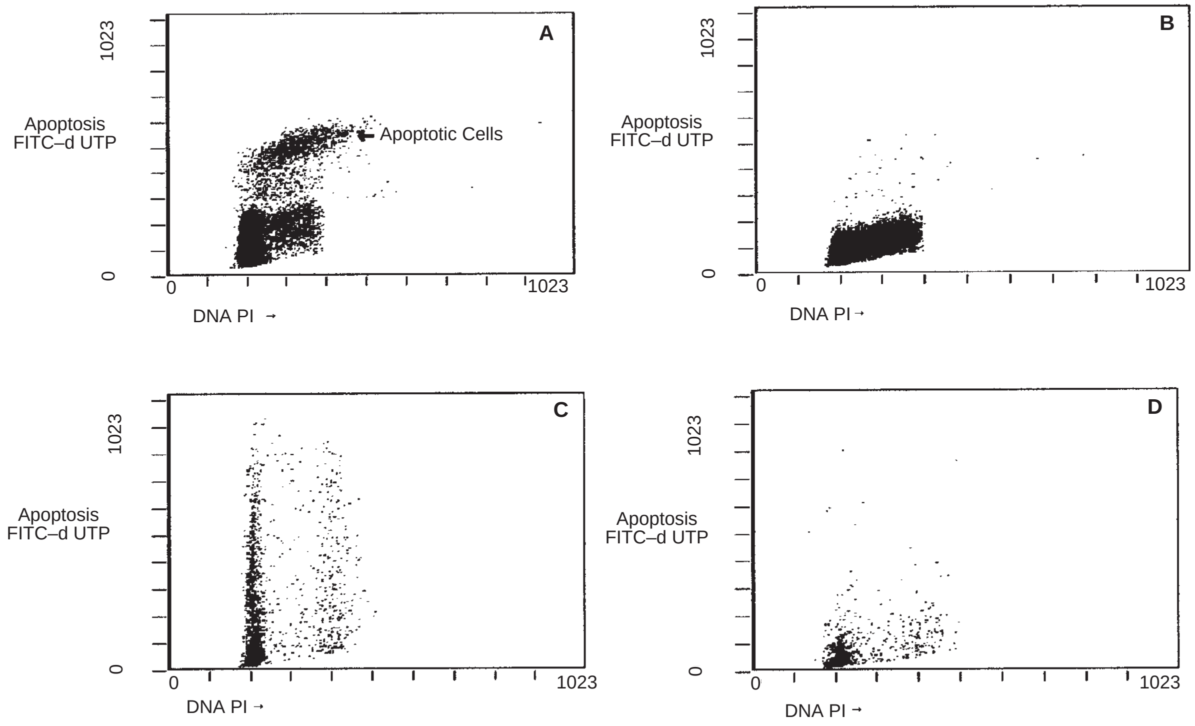

**Figure 2** Flow cytometric dot-plots of apoptosis vs DNA, for: (**A**) positive control cells; (**B**) negative control cells; (**C**) sample of colorectal mucosa; (**D**) sample colorectal cancer

Bedfordshire, UK) and mechanically disaggregated by passing through a fine wire mesh (~50 µm), as previously described (Brotherick et al, 1995). The resulting single-cell suspension was centrifuged at 400 ***g*** and the cell pellet resuspended in 10 ml of Isoton II. The cell concentration was assessed by haemocytometer and diluted to approximately $1 \times 10^6$ cells per ml. The cell suspension was divided into two equal portions, one for assessment of cellular DNA content and cell cycle phase fractions and one for the apoptosis assay. We have previously used cytokeratin gating in disaggregated breast cancers after saponin treatment to render cells permeable to antibodies (Brotherick et al, 1998). However, cytokeratin gating and assay of apoptosis in the same sample by the method of the present study is not possible. The APO-Direct kit (Pharmingen) provides a fluorescein isothiocyanate (FITC)–dUTP label that identifies DNA strand-breaks. The preparation method requires fixation and permeabilization of cells, which precludes reliable assay of cytoplasmic markers. Furthermore, the FITC–dUTP of the APO-Direct kit and commercially available labelled anticytokeratin antibodies have emission spectra of the same wavelength and cannot be assessed in the same sample. We therefore used singlet discrimination gating method only, to focus on cells of interest. This technique allows exclusion of cell doublets, triplets, aggregates or clumps. The total tumour cell population was assayed in this study.

### Calibration of flow cytometer

Flow cytometry was carried out using a FACScan (Becton Dickinson, Oxford, Oxfordshire, UK). Linearity of the amplifier and FACScan settings for fluorescence channel 1 (FL1, green) and 2 (FL2, red) were checked using standardized beads. Healthy human lymphocytes were isolated from peripheral blood and suspended in medium at $10^6$ cells per ml. These provided a normal control diploid cell population and were used for standardization of instrument settings. Aliquots (200 µl) were incubated at 4°C for 1 min with 10 µl of propidium iodide (PI, 0.25 mg $ml^{-1}$; Sigma) in 4.5% (v:v) Triton X-100 (non-fluorescent cytometry grade; BDH, Poole, Dorset, UK). RNAase (Sigma) 1 mg $ml^{-1}$ was added to remove cellular RNA, which may produce an associated fluorescent signal. Fluorescence of the nuclei was measured in the red spectrum using a 630 nm filter on the fluorescence 2 photomultiplier tube of the instrument. Linear amplification was from prestored instrument settings. The G0/G1 peak of freshly isolated lymphocytes was adjusted in the fluorescence 2 red spectrum to channel 100.

### Assay of tumour and mucosal cellular DNA content

Cell suspensions were diluted to $1 \times 10^6$ cells per ml, processed and analysed as above to assay DNA content. Then, $1 \times 10^5$ events were recorded for each specimen.

### Data analysis

DNA histograms were constructed and cell cycle phase fractions, and the presence of aneuploidy were assessed using Multicycle AV (Coulter) computer software, using previously described methods (Herman, 1992). DNA aneuploidy was identified only when two or more distinct (bimodal) peaks were present (Shankey et al, 1993). The percentage of cells in S phase was taken as an index of proliferation, in both the diploid and the aneuploid cell population, when present.

*© 1999 Cancer Research Campaign*

### Assessment of apoptosis

In cells undergoing apoptosis, DNA is degraded by nucleases with exposure of multiple 3´-hydroxyl termini. These exposed termini may be detected by the TUNEL (terminal deoxynucleotidyl transferase-mediated dUTP nick end-labelling) assay which identifies exposed termini. This method has been adapted for flow cytometry by Li et al (1995), to provide a quantitative assay of apoptosis.

Cell suspensions were centrifuged at 400 ***g*** and the supernatants discarded. Cells were fixed by incubation with 5 ml of 1% paraformaldehyde in Isoton II at 4°C for 15 min. The suspensions were centrifuged, the supernatants discarded and the cells washed with Isoton II twice. Five millilitres of ice-cold 70% (v/v) ethanol was added to each sample, which were then stored at –20°C overnight. DNA strand-breaks consistent with apoptosis, were assayed by TUNEL labelling, using the APO-DIRECT™ kit (Pharmingen, San Diego, CA, USA) which includes positive (i.e. apoptotic) and negative (i.e. non-apoptotic) control cell samples, prepared from the HL-60 human lymphoma cell line. Apoptosis was induced in the positive control cell line by treatment with Camptothecin. Negative control cells were untreated.

Suspensions of positive and negative control cells, mucosal and tumour cells were spun at 1000 ***g*** and the supernatants discarded, then washed twice in buffered saline. A staining solution was prepared using kit reagents, with terminal deoxynucleotidyl transferase (TdT) and fluorescein-tagged deoxyuridine triphosphate nucleotides (FITC-dUTP) in buffered saline. Fifty microlitres of the above solution was added to each cell pellet, vortexed gently then incubated at 37°C for 3 h. Buffered saline rinse (1 ml) was added to each suspension, spun at 1000 ***g*** and the supernatant discarded. The rinse was repeated, then samples were resuspended in a 0.5 ml solution of PI (0.01 mg $ml^{-1}$; Sigma) and RNAase (0.05 mg $ml^{-1}$; Sigma). The cells were incubated at room temperature for 30 min, then subjected to analysis of fluorescence by flow cytometry. A FACScan flow cytometer (Becton Dickinson) was used to measure the fluorescence of $1 \times 10^5$ cellular events. Nuclear DNA content was assayed on the fluorescence 2 setting while FITC-labelled DNA breaks were assayed on fluorescence 1.

### Flow cytometry analysis

Fluorescence data were analysed using LYSYS II software (Becton Dickinson). Nuclear doublets and other multiples were excluded from analysis by setting a gate using a dot-plot of fluorescence 2 width against fluorescence 2 area. For each sample, a histogram of fluorescence 1 was constructed. A marker was placed on the histogram for the negative control at the 97th percentile of fluorescence, and the value of this level of fluorescence recorded. A marker at this same level was applied to the other histograms, and the percentage of cells above this level calculated. This percentage of cells minus 3% was taken as the proportion of positive cells.

### Immunohistochemistry

Thirty-seven paraffin-embedded tumour specimens were available for immunohistochemistry. Sections of 5-µm thickness were cut and mounted on glass microscope slides, previously coated with 3-aminopropyl triethoxy-silane. Sections were dewaxed, soaked in 0.5% hydrogen peroxide in methanol and washed in cold water. Antigen retrieval was performed by heating in a pressure cooker for 2 min in citrate buffer (pH 6.0) at boiling point. Following a 5-min rinse in 0.005 M Tris-buffered saline (TBS), sections were covered with normal rabbit serum diluted 1:10 in TBS (NRS). Excess serum was removed and the sections were incubated consecutively with three antisera for 30 min, with $2 \times 5$ min washes in TBS after each, viz. (i) Novocastra DO7 monoclonal mouse anti-human antibody to p53 protein (NCL-p53-DO7) diluted 1:50 with NRS; (ii) Dako biotinylated rabbit anti-mouse antibody (E0354) diluted 1:500 with NRS; and (iii) streptavidin with biotinylated horseradish peroxidase (Dako streptABComplex/HRP), diluted 1:100 with NRS. Sections were incubated for 1 h. Peroxidase activity was developed with 3,3′-diaminobenzidine followed by a rinse in water. Haematoxylin was used as a counterstain and the sections were dehydrated and mounted in DePeX mounting medium (BDH). Sections were examined by light microscopy and the proportion of epithelial cells with nuclear staining calculated for each tumour by one observer (DSW). Tumours with over 50% nuclear staining were categorized as showing significant p53 accumulation in accordance with previous definitions (Manne et al, 1997).

### Statistical analysis

Descriptive statistics included the mean ± standard deviation (s.d.). The Wilcoxon matched pairs signed-rank test and the Mann–Whitney *U*–Wilcoxon rank sum *W*-test were used to assess cell cycle and apoptosis data in mucosal and tumour specimens. The Pearson's correlation test was used to investigate the relationship between proliferation and apoptosis.

### Ethics

Ethical approval for the study was granted by Ethical Committees of Newcastle University and South Tees Acute Hospitals NHS Trust, and by the Tayside Committee for Medical Ethics.

### RESULTS

### Clinical data

Forty-two patients were recruited into the study, 26 male and 16 female, with a mean age of 68 (range 49–79). Twelve tumours were right-sided and 30 left-sided, of which 20 were from the rectum.

### DNA content, cell proliferation and ploidy

All tumours contained diploid cell populations, while 11 carcinomas also included aneuploid cell populations. The proportions of cells in DNA S phase were similar in diploid (15.5 ± 12.9%) and aneuploid (15.6 ± 13.0) tumour cell populations. Tumour cell DNA S phase fraction was greater than that of normal mucosa (10.3 ± 6.6%; $P < 0.02$, by Wilcoxon matched pairs signed-rank test); (Figure 1A).

### Apoptosis

In the positive control HL-60 lymphoma line, 30% cells showed DNA strand-breaks indicative of apoptosis, consistent with suppliers findings. Apoptosis was found in 0–57% of cells in tumours and normal mucosa samples. A greater proportion of normal mucosa cells showed positive TUNEL labelling than

*© 1999 Cancer Research Campaign*

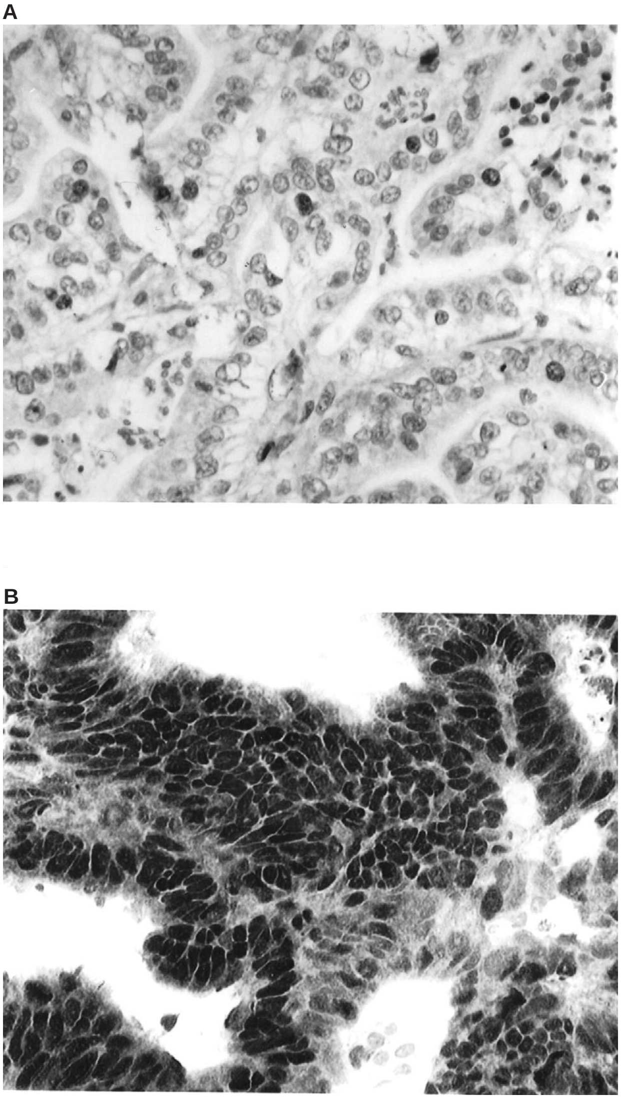

**Figure 3** (**A**) Colorectal carcinoma showing negative immunohistochemistry for p53. (**B**) p53 accumulation in a colorectal carcinoma, identified by > 50% nuclear staining. Immunohistochemistry used DO7 anti-human p53 antibody (Novocastra)

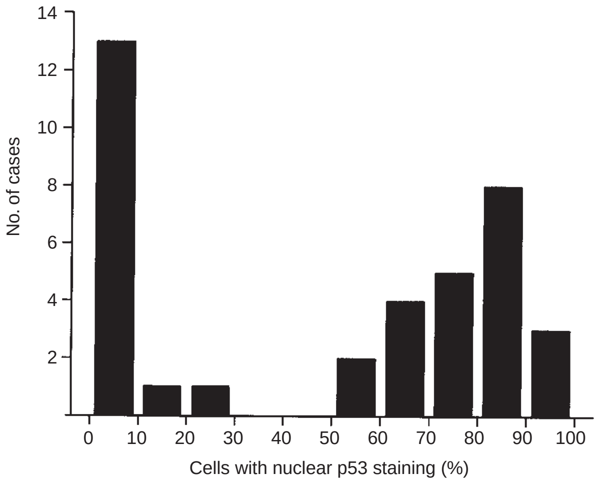

**Figure 4** Histogram showing the numbers of cancers with different proportions of nuclei with detectable p53

tumour cells (28.7 ± 17.9% mucosa vs 18.5 ± 17.0% tumours; $P < 0.01$, by Wilcoxon matched pairs signed-rank test) (Figure 1B). Representative flow cytometric dot-plots of apoptosis, plotted against DNA content, are shown in Figures 2 A–D.

### Relationship between proliferation and apoptosis

A significant relationship was observed between apoptosis identified by TUNEL and cell cycle DNA S phase fraction, in normal mucosa ($r = 0.8$; $P < 0.05$, Pearson's correlation test). No relationship was observed in tumour samples (Figure 1 C, D).

### Tumour stage

Three tumours were Dukes' stage A, 24 stage B and 15 C. No relationship was observed between tumour stage, apoptotic proportion or tumour cell S phase fraction.

### p53 immunohistochemistry

In 13 tumours, p53 immunostaining was undetected or involved < 10% cells, while in two tumours, staining involved 10–30% cells. p53 accumulation, defined as > 50% nuclear staining for p53, was detected in 22/37 (59%) carcinomas (Figure 3 A, B and Figure 4). Apoptosis was reduced in tumours showing p53 accumulation, compared to both p53-negative tumours and non-malignant mucosa (14.8 ± 15% p53 accumulation vs 26.3 ± 18% p53-negative tumours; $P < 0.05$ and 28.7 ± 17.9% mucosa; $P < 0.05$ both by Mann–Whitney *U*–Wilcoxon rank sum *W*-test). Tumours without p53 accumulation had apoptotic proportions indistinguishable from mucosal samples. p53 accumulation was unrelated to ploidy, DNA S phase fraction or Dukes' stage (Table 1).

### DISCUSSION

Mutation of the tumour suppressor p53 gene is a non-essential event during human colorectal carcinogenesis (Baker et al, 1990) and incurs loss of G1 checkpoint control to allow DNA repair (Kastan et al, 1991) or initiate apoptosis (Shaw et al, 1992) in response to DNA damage. These mechanisms may induce genomic instability (Bischoff et al, 1990) and acquisition of the multiple 'hits' required for cancer development (Nowell, 1976). In established cancers, p53 inactivation is commonly associated with intracellular p53 protein accumulation (Hall and Lane, 1994). The present study has shown p53 accumulation in 59% of colorectal tumours, in accord with previous findings (Kang et al, 1997; Ahnen et al, 1998).

Optimal regulation of proliferation or cell death is central to tissue integrity. This study found a significant relationship between DNA S phase fraction and apoptosis indicative of growth homeostasis, in normal human colorectal mucosa. No such relationship was found in the colorectal cancers. When all cancers were considered, the proportion of cells undergoing apoptosis was lower while the fraction in DNA synthesis phase was higher than in normal mucosa. On further analysis, however, it was found that the difference in apoptosis was largely confined to cancers with p53 accumulation. DNA S phase fraction was unrelated to p53. The findings point to a growth dysequilibrium associated with p53 accumulation.

 *© 1999 Cancer Research Campaign*

Clonal divergence occurs within human colorectal cancers. Mutant p53 promotes genomic instability (Bischoff et al, 1990) and may facilitate ploidy change (Carder et al, 1993). Previous studies have shown that inactivation of p53 precedes, and may facilitate, emergence of multiple cell populations which differ in DNA ploidy (Carder et al, 1995). The findings of the present study, that p53 accumulation confers growth dysequilibrium and selection pressure, may enable expansion of these unstable clones. An association between ploidy and p53 status in colorectal cancer has been confirmed by previous studies. Attallah et al have shown DNA aneuploidy in 75% of p53-positive colorectal tumours, compared to only 64% in p53-negative cancers (Attallah et al, 1997). Tumour heterogeneity of aneuploidy has been described, and sampling at a single site could produce an underestimation of the true extent. This factor could account for the lower incidence of aneuploidy in the present study, than reported by previous authors (Carder et al, 1995; Attalah et al, 1997). The number of aneuploid tumours in the present study was too small for investigation of any relationship to p53 status.

Given the association between p53 accumulation, genomic instability (Carder et al, 1993) and growth dysequilibrium shown in this study, an association between p53 accumulation and prognosis could be anticipated. In this context, previous studies have reported different findings in colorectal cancer. Lehy et al reported that immunohistochemical overexpression of p53 protein was an independent prognostic indicator of poor survival in a study of 66 patients (Lehy et al, 1996). Conversely, Mulder et al, in a study of 109 colorectal carcinomas, found that immunohistochemical p53 expression was not a useful marker for long-term prognosis (Mulder et al, 1995). In the present study, clinical follow-up was for a short interval only and accurate prognostic assessment was not possible. We found no relationship between p53 accumulation and Dukes' stage, however.

In summary, this study has confirmed loss of growth homeostasis in colorectal cancer. p53 accumulation is accompanied by reduced apoptosis which may confer a growth advantage. Further insight is necessary concerning p53-dependent growth regulation and p53-independent mechanisms of tumour behaviour, including invasion or metastatic potential, which may also influence prognosis.

### ACKNOWLEDGEMENTS

The authors gratefully acknowledge the assistance of Dr B Angus at the Department of Pathology at Newcastle University, Dr R Jones at the Department of Pathology, Middlesborough General Hospital, Mr WJ Cunliffe and Mr DA Browell of Queen Elizabeth Hospital, Gateshead, Mr PJ Hainsworth of Freeman Hospital and Mr JS Varma of The Royal Victoria Infirmary, Newcastle upon Tyne.

This project was funded by a grant from the Newcastle Hospitals Special Trustees and by South Tees Acute Hospitals NHS Trust.

### REFERENCES

Ahnen DJ, Feigl P, Quan G, FenoglioPreiser C, Lovato LC, Bunn PA, Stemmerman G, Wells JD, Macdonald JS and Meyskens FL (1998) Ki-ras mutation and p53 overexpression predict the clinical behaviour of colorectal cancer: a southwest oncology group study. *Cancer Res* **58**: 1149–1158

Attallah AM, Elhak NG, Nasif WA, Tabll A and Ezzat F (1997) Detection of p53 protein overexpression and DNA ploidy analysis in colon cancer. *Hepato-gastroenterology* **44**: 1595–1601

Baker SJ, Markowitz S, Fearon ER, Willson JKV and Vogelstein B (1990) Suppression of human colorectal-carcinoma by wild-type-p53. *Science* **249**: 912–915

Bargonetti J, Friedman PN, Kern SE, Vogelstein B and Prives C (1991) Wild type but not mutant p53 immunopurified proteins bind to sequences adjacent to the SV40 origin or replication. *Cell* **65**: 1083–1091

Bischoff FZ, Yim SO, Pathak S, Grant G, Sicilano MJ, Giovanella BC, Strong LC and Tainsky MA (1990) Spontaneous abnormalities in normal fibroblasts from patients with Li–Fraumeni cancer syndrome: aneuploidy and immortalization. *Cancer Res* **50**: 7979–7984

Brotherick I, Shenton BK, Angus B, Waite IS, Horne CHW and Lennard TWJ (1995) A flow cytometric study of c-erbB-3 expression in breast cancer. *Cancer Immunol Immunother* **41**: 280–286

Brotherick I, Browell DA, Shenton BK, Egan M, Cunliffe WJ, Webb LA, Lunt LG, Young JR and Higgs MJ (1998) The effect of 3-week tamoxifen treatment on oestrogen receptor levels in primary breast tumours: a flow cytometric study. *Br J Cancer* **77**: 1657–1660

Carder P, Wyllie AH, Purdie CA, Morris RG, White S, Piris J and Bird CC (1993) Stabilized p53 facilitates aneuploid clonal divergence in colorectal cancer. *Oncogene* **8**: 1397–1401

Carder PJ, Cripps KJ, Morris R, Collins S, Bird CC and Wyllie AH (1995) Mutation of the p53 gene precedes aneuploid clonal divergence in colorectal cancer. *Br J Cancer* **71**: 215–218

Clarke AR, Purdie CA, Harrison DJ, Morris RG, Bird CC, Hooper ML and Wyllie AH (1993) Thymocyte apoptosis induced by p53-dependent and independent pathways. *Nature* **362**: 849–851

El-Deiry WS, Tokino T, Velculescu VE, Levy DB, Parsons R, Trent JM, Lin D, Mercer WE, Kinzler KW and Vogelstein B (1993) WAF1, a potential mediator of p53 tumour suppression. *Cell* **5**: 817–825

Fearon ER and Vogelstein B (1990) A genetic immunohistochemistry model for colorectal tumourigenesis. *Cell* **61**: 759–767

Hall PA and Lane DP (1994) p53 in tumor pathology: can we trust immunohistochemistry revisited. *J Pathol* **172**: 1–4

Herman CJ (1992) Cytometric DNA analysis in the management of cancer. Clinical and laboratory considerations [Review]. *Cancer* **69**: 1553

Kang SM, Maeda K, Onoda N, Chung YS, Nakata B, Nishiguchi Y and Sowa M (1997) Combined analysis of p53 and vacular endothelial growth factor expression in colorectal carcinoma for determination of tumour vascularity and liver metastasis. *Int J Cancer* **74**: 502–507

Kastan MB, Onykwere O, Sidransky D, Vogelstein B and Craig RW (1991) Participation of p53 protein in the cellular response to DNA damage. *Cancer Res* **51**: 6304–6311

Kobayashi M, Watanabe H, Ajioka Y, Yoshida M, Hitomi J and Asakura H (1995) Correlation of p53 protein expression with apoptotic incidence in colorectal neoplasia. *Virchows Arch* **427**: 27–32

Langlois NE, Lamb J, Eremin O and Heys SD (1997) Apoptosis in colorectal cancer occurring in patients 45 years and under: relationship to prognosis, mitosis and immunohistochemical demonstration of p53, c-myc and bcl-2 protein products. *J Pathol* **182**: 392–397

Lanza G, Maestri I, Dubini A, Gafa R, Santini A, Ferretti S and Cavazzini L (1996) P53 expression in colorectal cancer – relation to tumor type, DNA-ploidy pattern, and short-term survival. *Am J Clin Pathol* **105**: 604–612

Lehy DT, Salman R, Mulcahy H, Sheahan K, O'Donoghue DP and Parfrey NA (1996) Prognostic significance of p53 abnormalities in colorectal carcinoma detected by PCR-SSCP and immunohistochemical analysis. *J Pathol* **180**: 364–370

Li X, Traganos F, Melamed MR and Darzynkiewicz Z (1995) Single step procedure for labelling DNA strand breaks with fluorescein- or BODIPY-conjugated deoxynucleotides. Detection of apoptosis and bromodeoxyuridine incorporation. *Cytometry* **20**: 172–180

Manne U, Myers RB, Moron C, Poczatek RB, Dillard S, Weiss H, Brown D, Srivastava S and Grizzle WE (1997) Prognostic significance of Bcl-2 expression and p53 nuclear accumulation in colorectal adenocarcinoma. *Int J Cancer (Pred Oncol)* **74**: 346–358

Mulder JWR, Baas IO, Polak MM, Goodman SN and Offerhaus GJA (1995) Evaluation of p53 protein expression as a marker for long-term prognosis in colorectal carcinoma. *Br J Cancer* **71**: 1257–1262

Nowell PC (1976) The clonal evolution of tumour cell populations. *Science* **194**: 23–28

Purdie CA, O'Grady J, Piris J, Wyllie AH and Bird CC (1991) P53 expression in colorectal tumors. *Am J Pathol* **138**: 807–813

Save V, Nylander K and Hall PA (1998) Why is p53 protein stabilised in neoplasia? Some answers but many more questions! *J Pathol* **184**: 348–350

Scott N, Sagar P, Stewart J, Blair GE, Dixon MF and Quirke P (1991) p53 in colorectal cancer: clinicopathological correlation and prognostic significance. *Br J Cancer* **63**: 317–319

© 1999 Cancer Research Campaign 

Shankey TV, Rabinovitch PS, Bagwell CB, Bauer KD, Duque RE, Hedley DW, Mayall BH, Cox C and Wheeless L (1993) Guidelines for the implementation of clinical DNA cytometry. *Cytometry* **14**: 472–477

Shaw P, Bovey R, Tardy S, Sahli R, Sordat B and Costa J (1992) Induction of apoptosis by wild-type p53 in a human colon tumor-derived cell-line. *Proc Natl Acad Sci USA* **89**: 4495–4499

Tsujitani S, Shirai H, Tatebe S, Sugamura K, Ohfuji S, Gomyo Y, Maeta M, Ito H and Kaibara N (1996) Apoptotic cell death and its relationship to carcinogenesis in colorectal carcinoma. *Cancer* **77**: 1711–1716

*© 1999 Cancer Research Campaign*
